# Nanocone-Shaped Carbon Nanotubes Field-Emitter Array Fabricated by Laser Ablation

**DOI:** 10.3390/nano11123244

**Published:** 2021-11-29

**Authors:** Jiuzhou Zhao, Zhenjun Li, Matthew Thomas Cole, Aiwei Wang, Xiangdong Guo, Xinchuan Liu, Wei Lyu, Hanchao Teng, Yunpeng Qv, Guanjiang Liu, Ke Chen, Shenghan Zhou, Jianfeng Xiao, Yi Li, Chi Li, Qing Dai

**Affiliations:** 1 Tianjin Key Laboratory of Molecular Optoelectronic Sciences, Department of Chemistry, School of Science, Tianjin University & Collaborative Innovation Center of Chemical Science and Engineering (Tianjin), Tianjin 300072, China; zhaojz2020@nanoctr.cn (J.Z.); lvw0817@163.com (W.L.); 2 CAS Key Laboratory of Nanophotonic Materials and Devices, CAS Key Laboratory of Standardization and Measurement for Nanotechnology, CAS Center for Excellence in Nanoscience, National Center for Nanoscience and Technology, Beijing 100190, China; lizhenjun@nanoctr.cn (Z.L.); wangaw2020@nanoctr.cn (A.W.); guoxd@nanoctr.cn (X.G.); liuxc2019@nanoctr.cn (X.L.); tenghc2019@nanoctr.cn (H.T.); quyp2020@nanoctr.cn (Y.Q.); liugj20101120@163.com (G.L.); chenke@nanoctr.cn (K.C.); zhoushenghan@nanoctr.cn (S.Z.); xiaojf2020@nanoctr.cn (J.X.); 3 GBA Research Innovation Institute for Nanotechnology, Guangzhou 510700, China; 4 Department of Electronic and Electrical Engineering, University of Bath, Bath BA2 7AY, UK; mtc47@bath.ac.uk; 5 Center of Materials Science and Optoelectronics Engineering, University of Chinese Academy of Sciences, Beijing 100049, China; 6 Joint School of National University of Singapore and Tianjin University, Fuzhou International Campus, Tianjin University, Binhai New City, Fuzhou 350207, China

**Keywords:** nanocone array, field emission, carbon nanotubes, laser ablation

## Abstract

The nanocone-shaped carbon nanotubes field-emitter array (NCNA) is a near-ideal field-emitter array that combines the advantages of geometry and material. In contrast to previous methods of field-emitter array, laser ablation is a low-cost and clean method that does not require any photolithography or wet chemistry. However, nanocone shapes are hard to achieve through laser ablation due to the micrometer-scale focusing spot. Here, we develop an ultraviolet (UV) laser beam patterning technique that is capable of reliably realizing NCNA with a cone-tip radius of ≈300 nm, utilizing optimized beam focusing and unique carbon nanotube–light interaction properties. The patterned array provided smaller turn-on fields (reduced from 2.6 to 1.6 V/μm) in emitters and supported a higher (increased from 10 to 140 mA/cm^2^) and more stable emission than their unpatterned counterparts. The present technique may be widely applied in the fabrication of high-performance CNTs field-emitter arrays.

## 1. Introduction

Vertically aligned carbon nanotubes (VACNT) provide a much simpler, cheaper, as well as more scalable, manufacturable, and deployable platform upon which to build future electron sources. A variety of methods have come to the fore to achieve VACNT field-emitter arrays with well-defined patterns, which include the pre-patterning, commonly by nanostamping [1], electron beam lithography [2], or photolithography [3] of the catalyst layer. Although very successful, all these approaches are costly and require time-consuming additional process steps [4] before the CNT growth. Moreover, it is difficult to achieve large length-to-diameter ratios of >8, and it is not possible to realize more diverse shapes that are patterned in the out-of-plane direction, such as structures with nanoscale tips. Fundamentally new approaches are required to fabricate VACNT array with nanoscale tips (nanocone) that can be achieved at low cost and high throughput.

Post-growth, laser ablation patterning is one exciting solution that satisfies the above requirements. Laser processing is a technology that uses the interaction between laser and material to cut, weld, surface treatment, micromachining, and so on [5,6,7,8,9,10,11,12]. The photons of low wavelength ultraviolet (UV) light have high energy, and the single photons of high energy (>3 eV) can directly break (requires 3–10 eV) the chemical bonds of materials (photolytic process) [10]. This is a highly nonlinear process, which make it possible to process materials with sharp edges. In addition, the almost perfect absorption of VACNTs [13,14] for light in a wide wavelength range further reduces the heat-affected zone in the processing process [15]. These factors together make it possible for the laser to process in pattern micron or even nanoscale CNTs structures, which have high and stable current under low electric field intensity.

Here, to create functionally enhanced CNT arrays, we report an ultraviolet laser patterning technique that is capable of realizing a range of well-defined emitter morphologies by accurately adjusting the energy output state and action time of laser to VACNT. At the optimized condition, nanocone-shaped carbon nanotube arrays (NCNAs) were achieved. Laser-processed samples showed up to a 1 V/μm decrease in the turn-on electric field, more than a 97× increase in current density over the unpatterned counterparts, and improved stability (the current decay rate was reduced by more than 20%). To explore the impacts of the microscale geometry on the emission characteristics of the VACNT thin films, three-dimensional finite element simulations were also undertaken on unpatterned and patterned emitters.

## 2. Experimental

**Figure 1:**
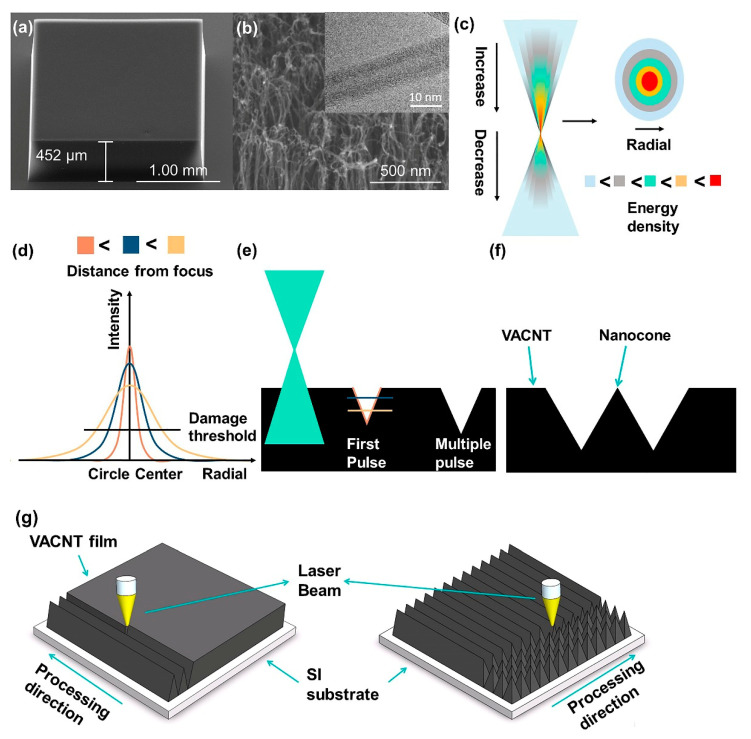
Schematic of the VACNT UV laser patterning process: (**a**) SEM images of a typical as-synthesized PECVD VACNT thin film (scale bar: 1 mm, Tilt: 45°). (**b**) High-resolution SEM image of the VACNT thin film upper surface highlighting the surface disorder and residual catalyst particles (scale bar: 500 nm, Tilt: 45°). Inset: High-resolution TEM of an individual as-grown PECVD CNT showing a wall thickness of approximately 0.12 nm, consisting of three graphitic side walls (scale bar: 10 nm). (**c**) Energy distribution diagram of the laser beam. (**d**) Gaussian distribution of laser beam energy. (**e**) The process of laser beam energy acting on VACNT to form its shape. (**f**) Nanocone and (**g**) Nanocone array formation process.

In brief, macroscale 2 mm × 2 mm square monoliths of VACNT thin films, which were ready for subsequent UV laser processing were synthesized on silicon substrate by PECVD (plasma-enhanced chemical vapor deposition) [11,16,17,18]. Respectively, as shown in Figure 1a,b, femtosecond laser processing has been demonstrated elsewhere though commonly at different wavelengths such as 800 nm and 1064 nm [19,20,21]; however, in the present work, a nanosecond ultraviolet laser processing platform (λ = 355 nm, Suzhou Delong laser Co. Ltd., Suzhou, China; FP-D-DZS-001) was used. AutoCAD (Version 2018, Autodesk, Inc., Mill Valley, CA, USA) software was used in this work to create DGW files required by the laser patterning system. The CAD drawings outline the path of the laser in two dimensions. The coordination of various parameters determines the width, depth, shape, continuity, and smoothness of the ablation groove, which together with the CAD drawings determines the shape of the VACNT created by the laser. (Supplementary Materials Figure S1 shows the arrays with high aspect ratio. Supplementary Materials Figure S2 shows CAD drawings and the corresponding processed shapes).

It has been shown elsewhere that micro and macro-scale geometries within CNT arrays enhanced their field electron emission performances [19,20,21]. Thus, here, we explored the use of the developed patterning technique to engineer new electron emission sources. Cubic arrays with sides of 20 μm and spacing of 40 μm were first fabricated on the VACNT film; then, we used this as a benchmark to continuously reduce the size of the tip and fabricated the cone arrays with the tip size of about 15 μm, 10 μm, 5 μm, 2.5 μm, and 500 nm on the VACNT film, which were all initially 608 ± 20 μm thick.

The effects of different laser processing parameters on the carbon nanotubes had been explored, including Raman spectroscopy as a function of laser power, which were also were characterized by X-ray photoelectron spectroscopy (XPS) to explore the impacts of carbon ablation on the VACNTs chemical composition and crystallography before and after processing.

Field-emission measurements were carried out in a custom-built vacuum chamber evacuated to a base pressure of <1 × 10^−7^ mbar. Measurements were conducted in diode mode. To minimize anode-induced arcing, the anode was formed from a 5 mm thick mechanically polished stainless-steel plate (surface roughness), with the cathode formed from either a processed or unprocessed VACNT thin film on the stainless-steel substrate, in which the distance between cathode and anode was 462 ± 20 μm. Voltages were swept from 0 to 10 kV with ∆V = 50 V and a dwell/step time of 1 s. A schematic diagram of the test equipment is in Supplementary Materials Figure S3.

A three-dimensional numerical calculation by Comsol Multiphysics software (Version 5.5, Comsol, Inc., Stockholm, Sweden) was carried out to verify the influence of field screen effect on carbon nanotubes cold cathode. (See Supplementary Materials for details of the simulation).

## 3. Results and Discussion

### 3.1. Laser Processing

In the present work, the nanosecond UV laser optical ablation system is employed, which represents a cheaper and more widely available means of accessing carbon ablation whilst benefitting from a known strong leading UV absorption edge within graphitic carbon nanomaterials [13,14,22]. This will be more conducive to the realization of industrialized mass production. Compared with infrared laser processing methods that use the thermal effects to burn out the VACNT [10,23,24], the high-energy ultraviolet photons are more likely to directly destroy the molecular bonds of the material and make the molecules separate from the original material [10]. In addition, the UV laser can achieve a smaller spot and smaller heat-affected zone, which means that it can achieve more sharp machining. The most important parameters in the processing include the lasing frequency (20–150 kHz), optical power (0–12 W), scanning speed (0.01–10,000 mm/s), scanning times, and the distance between the sample and the focus (the Supplementary Materials Figures S4 and S5 shows more details of laser machining).

Figure 1 shows the formation mechanism of the NCNA. As shown in Figure 1c, the maximum energy density and the minimum spot are obtained at the focus. After passing through the focus, the energy begins to diverge, and the spot slowly becomes larger. However, due to the propagation loss of light in the air, the energy distribution of the two positions symmetrical about the focus is not the same. In the radial direction of the spot, the energy of the spot center is the highest and decreases to 0 along the radial direction. The energy distribution law of the laser conforms to the Gaussian distribution, that is, the curve shown in Figure 1d. Therefore, when the laser beam hits the VACNT, the energy impact on the upper surface of the VACNT should conform to the energy distribution corresponding to the spot.

After the first pulse, the CNT in the area where the energy is greater than the damage threshold (ablation threshold of the VACNTs approximately 50 mJ·cm^−2^ [25]) of VACNT will be removed, leaving a shape similar to that above the damage threshold of the Gaussian curve on VACNT (it is assumed that the components of energy points in all directions are almost uniform), which can be approximated as a triangle, as shown in Figure 1e. The second pulse will continue to act on VACNT along the shape generated by the first pulse. Since the laser beam energy has the characteristics of Gaussian distribution, at this time, the energy impact on each point on the edge of the shape formed by the first pulse will follow different Gaussian distribution curves. The formation of the final shape of the groove processed on VACNT is the result of the accumulation of multiple energy points with Gaussian distribution following different characteristics in space. According to our research on laser processing parameters (Supplementary Materials) and the previous reports of Tang [20] et al., after multiple pulses, the shapes of grooves processed on VACNT are not the same, one of which is triangular. The nanoscale edges can be obtained by accurately controlling the position of two adjacent triangular machining grooves, as shown in Figure 1f. Thus, NCNA can be obtained by continuous transverse and longitudinal scanning of VACNT, as shown in Figure 1g.

According to the above discussion, the change of focus value will make the upper surface of VACNT in Gaussian energy distribution have different characteristics, which will change the width and depth of the area that can be damaged by the first pulse energy and then affect the shape of the machining groove. The increase (decrease) of power will not change the energy distribution characteristics of the upper surface of VACNT but will increase (decrease) the energy as a whole; that is, in the same Gaussian distribution characteristics, a wider (narrower) area will reach the damage threshold of VACNT, thus affecting the length and width (or the size and shape of the area affected by each pulse) of the machining groove. The frequency mainly changes the number of pulses per unit time. Together with the scanning speed and scanning times, it controls the number of pulses in the unit area. The scanning speed and scanning times jointly control the residence time of the laser beam on the VACNT. The difference is that if the scanning times are fixed, adjusting the laser beam residence time by adjusting the scanning speed means that the thermal effect of the laser on the VACNT is more continuous. On the contrary, if the scanning speed is fixed, adjusting the scanning times may reduce the accumulation of thermal effects in a certain area. To some extent, laser frequency, scanning speed, and scanning times also affect the shape of the ablated groove. Of course, their cooperation is particularly important.

### 3.2. Impacts on the Structure and Surface Pre- and Post-Processing

**Figure 2:**
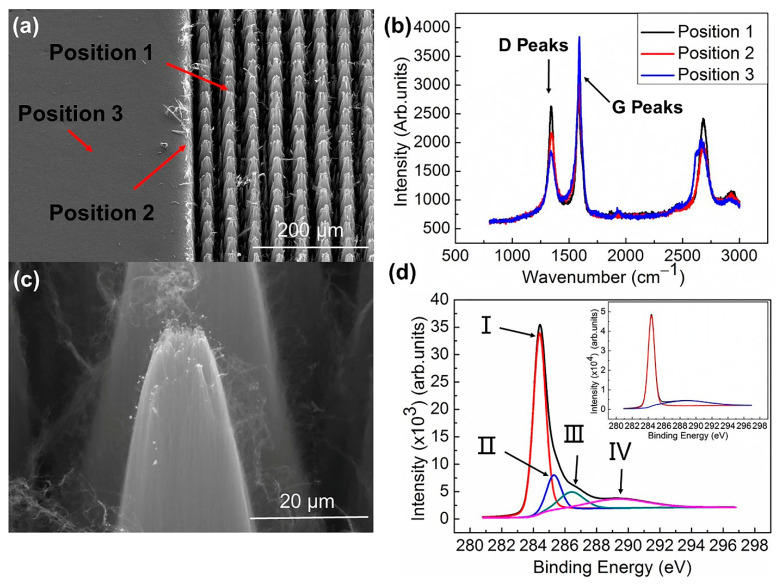
(**a**) A typical SEM image of a patterned VACNT sample where the right side has been processed. Position 1 is the top of the laser processed array, position 2 is the junction of the processed position and the unprocessed position, and position 3 is the unprocessed position. (**b**) Corresponding Raman spectra at the positions 1, 2, and 3. (**c**) New large exotic nanoparticles of Al and Fe on the processed VACNT. (**d**) The C 1s XPS peaks for processed VACNT.

To further explore the chemical and physical impacts of the UV laser ablation on the processed VACNT, Raman spectra and XPS were undertaken pre and post-processing. Figure 2a shows the VACNT with only the right half processed. Positions 1, 2, and 3 correspond to the processing position, the junction of processing and unprocessed position, and the unprocessed position, respectively. The Raman test results at positions 1, 2, and 3 have been shown in Figure 2b. The D peak was mainly induced by structural defects of CNT, amorphous carbon, or contaminants, and the G peak represents the degree of SP2 hybridization in CNT [26]. From position 1–3, the intensity of the D peak increased gradually and the G peak decreased gradually, with the ID/IG values of positions 1, 2, and 3 being 0.93, 0.70, and 0.48. It indicated that those positions (position 1) processed had more structural defects than the unprocessed sites (position 3). During processing, high-energy ultraviolet photons might directly degrade the chemical bonds within carbon nanotubes; at the same time, the heat generated during processing may also destroy the chemical bond and form pyrolytic carbon, broken chemical bonds combined with other elements in the air, resulting in more defects and amorphous carbon [27]. Position 2 is less affected than position 1, so its ratio of ID to IG is slightly less than that of position 1. We also tested the ID/IG values of groove edges with different powers; they were all higher than the unprocessed position. This may be because in the process of UV laser processing, the number of defects caused by photon direct cutting bonding is greater than that caused by the thermal ablation effect, which makes the value of ID/IG near the ablation groove higher (see Supplementary Materials Figures S6 and S7).

Interestingly, we found some nanoparticles at the top of the processed CNTs and the junction of the processed and the unprocessed regions, as shown in Figure 2c. This is likely residual Fe and Al catalyst materials, which both, when optically excited, reacted with the VACNTs and the ambient gaseous environment to form new large exotic nanoparticles [28]. The optical coupling to these metallic nanoparticles likely enhanced the optical coupling to the VACNT system, allowing us to access low-power densities. This shows that there is a thermal effect in the UV laser processing of carbon nanotubes. In some previous reports using femtosecond laser [19], the ablated edge has fewer defects than unprocessed. It has been shown elsewhere that temperatures of up to 1000 °C can be effective at driving the graphitization of otherwise defective nanocarbons due to their small size [29]. However, there are also some findings of Hai et al. [23] which have shown that elevated temperatures over 525 °C can result in the combustion of carbon nanotubes that further lead to material removal due to CO and CO_2_ formation. It was proposed that the high temperature generated in the laser processing helps to burn amorphous carbons away [30].

XPS showed that in addition to C, there is a small amount of O in the raw VACNT. Compared with the raw VACNT, the O concentration in the UV laser-processed VACNT was significantly increased, and there is a small amount of N and other trace elements in the air. The C 1s, O 1s, and N 1s peaks of processed VACNT and the C 1s and O 1s peaks of unprocessed VACNT are clearly visible in the XPS survey scan spectrum (see Supplementary Materials Figure S8a). The O concentration in the raw VACNT is 0.97%; the O and N concentrations in the processed VACNT are 7.54% and 0.88%. We examined the C 1s XPS peaks for processed (Figure 2d) and raw VACNT (see Supplementary Materials Figure S8b). As shown in Figure 2d, peak I represents the graphite-like C–C bonds at 284.4 eV, the peaks at 285.3 eV (II) and 286.4 eV (III) are the different types of the C–N bones, peak II corresponds to the SP2 trigonal C–N bonding, and peak III corresponds to SP3 tetrahedral C–N bonding, while the last peak (IV) at 289.3 eV is attributed to C–O type bonds [31,32,33,34]. The C 1s peaks of the raw VACNT only show graphite-like C–C bonds and C–O type bonds. O in the unprocessed samples may be due to the oxidation of the VACNTs during post-growth air exposure [32].

The appearance of N and other trace elements, as well as the increase in O content in the samples after UV laser processing is likely due to reactions with some elements in the air combining with C atoms with incomplete chemical bonds after the UV photons cut off the chemical bonds and finally doped into VACNT to form some vacancy-related defects. In terms of electronic state, the presence of surface-localized oxygen molecules has played a positive role in improving the field emission performance due to the generation of new states induced by the O_2_ [35]. N doping is commonly beneficial; it enhances electron emission because doped nitrogen atoms could replace carbon atoms in carbon nanotubes and therefore increase the electronic density [35]. In terms of structure, the O and N all had been shown to improve electron emission because they can usually make carbon nanotubes form open edges, and also the engineered tip morphologies provide more small emission tips on the surface of carbon nanotubes [31,36,37]. Our findings suggest that the 5% composition of the identified N and O tends to vary between samples, which is likely due to the energy sensitivity and inevitable process variability of the bond formation (XPS survey scan spectrum and the C1s XPS peaks of another sample showed in Supplementary Materials Figure S8c,d).

### 3.3. Field Emission Properties as a Function of Conical Tip Radius of Curvature

**Figure 3:**
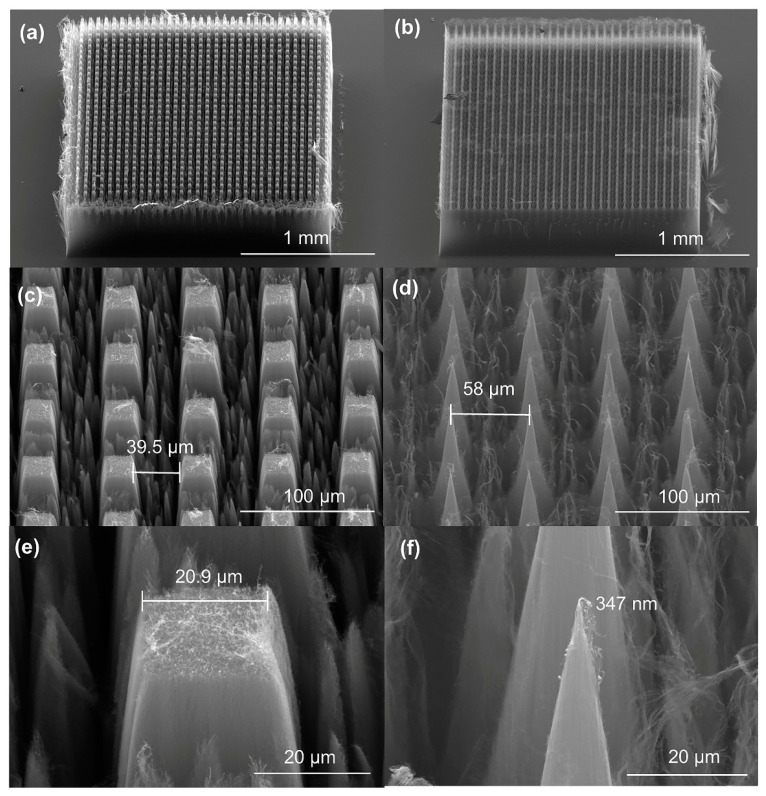
(**a**) A typical UV laser-processed cuboid array with a side length of about 20 μm and (**b**) 500 nm consisting of 1089 elements. (**c**) An enlarged view of the processed array showing the formed tips of about 20 μm and (**d**) about 500 nm. (**e**) A single tip in the array with a radius of curvature of 20.9 μm and (**f**) 347 nm.

To study the influence of the processed VACNT tip radius of curvature on their field emission properties, cuboid arrays with a side length of about 20 μm and spacing of about 40 μm were firstly fabricated and UV laser processed to create tips of the engineered radius of curvature of 20 µm, 10 µm, 5 µm, 2.5 µm, and 500 nm. Figure 3a–d shows the morphology arrays with a side length of about 20 μm and 500 nm; Figure 3e,f are the enlarged view of their local area and show one of the arrays. Photographs of other tip sizes and some typical samples are included in the Supplementary Materials Figure S9.

Due to the slight jitter of the carbon nanotube film fixed on the sample table by vacuum suction during the processing, the shape and size of each small array may be slightly different, which is within the allowable error range. Our electron microscopy studies highlighted several small (<50 nm diameter) CNT emitters within the processed arrays, which we believe positively contribute to the enhanced electron emission. Further work is ongoing to investigate the kinetics associated with their formation so as to derive further control in their placement and formation. Meanwhile, the emitters of some small carbon nanotubes are relatively chemically stable. (Please refer to Supplementary Materials Figure S10 for photos).

**Figure 4:**
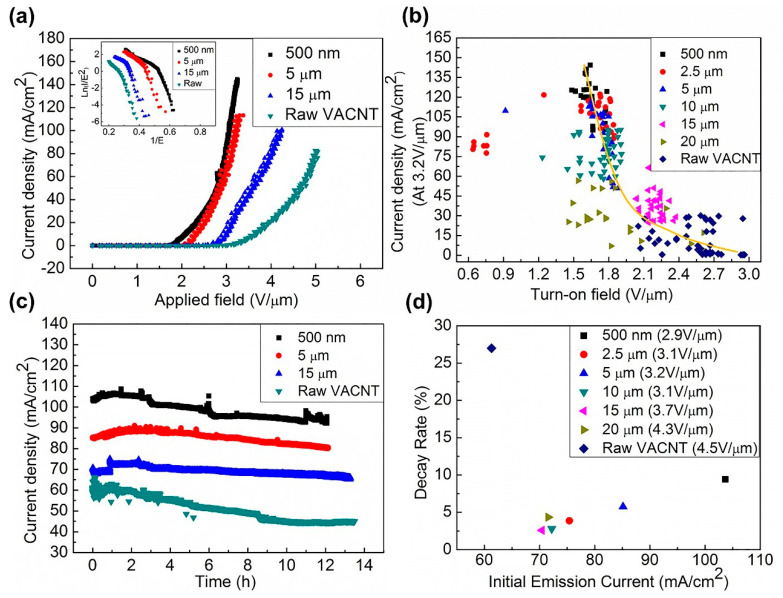
(**a**) Current density–applied field curves of the raw VACNT film; the cone arrays of the tip size are about 500 nm, 5 μm, and 15 μm, and the illustration is Forwer–Nordheim plots. (**b**) The statistical diagram of the relationship between the open field strength and the current density of the unprocessed samples and the cone array samples with different tip sizes. (**c**) Current–time stability curves of the raw VACNT film; the cone arrays of the tip size are 500 nm, 5 μm, and 15 μm. (**d**) The relationship between the current decay rate (12 h) and the initial current of raw samples and cone array samples with different tip sizes was studied. (The calculated emission is 4 mm^2^ from (**a**–**d**)).

To examine the impact of tip radius of curvature on the field electron emission performances, unprocessed and processed VACNT film cathodes were tested in a custom-build ultra-high vacuum environment; all tested samples had a similar height (608 ± 20 μm) and the same number of pillars. Figure 4a shows the current density as a function of the applied global electric field for a representative raw sample and cone arrays of tip sizes 500 nm, 5 μm, and 15 μm. The nanocone sample with a tip size of 500 nm had the best performance. Its turn-on electric field (defined as reported elsewhere, specifically as the electric field that stimulates an emission current of 1 μA) is 1.6 V/μm, which decreased by 1 V/μm. Its emission current density reached up to 144.5 mA/cm^2^ (the calculated emission area of the 500 nm nanocone array is 4 mm^2^) at the applied electric field of 3.2 V/μm. At the applied field of 3.2 V/μm, the current density is enhanced by 97 times compared with the unprocessed sample. If only considering the area of the tips, the current density of the nanocone array with a tip size of 500 nm is 2.703 × 10^6^ mA/cm^2^ (the calculated emission area of 500 nm nanocone array is 213.8 μm^2^), which are 1.814 × 10^6^ times higher than that of the raw sample. More than three samples for each tip diameter were tested, and the data were plotted in the J–E_on_ plot in Figure 4b. The observed variation in the measured performance of the processed emitters is due to the relative youthfulness of the ablation patterning process, which induces observed marginal morphological sample-to-sample variations. As FE is particularly sensitive to surface chemistry as well as emitter geometry, such small variations result in somewhat larger observed variations in the J–E_on_ data. In what follows, we take the average value for each tip set of cone array for comparison.

Our post-emission SEM findings suggested that laser processing tended to marginally compromise the degree of adhesion of the VACNTs with the underlying substrate. Figure 4c shows the DC temporal stability, conducted over 12 h, of these VACNT samples. Compared with the unpatterned samples, nanocone-patterned samples were noted to be more stable. Figure 4d shows the relationship between the initial current and the decay rate of raw VACNT and cone arrays with different tip sizes. It can be seen that the UV laser-processed arrays generally have higher stability and simultaneously achieve higher total current and lower current decay. We also found under the same experimental conditions that the field emission impact damage observed on the samples of the tested UV laser-processed samples was significantly smaller than that of the unprocessed samples, which was due to the lower driving voltage. (See Supplementary Materials Figure S11 for pre and post-field emission material characterization).

### 3.4. Simulation of Electric Field Intensity with a Different Tip Radius of Curvature

**Figure 5:**
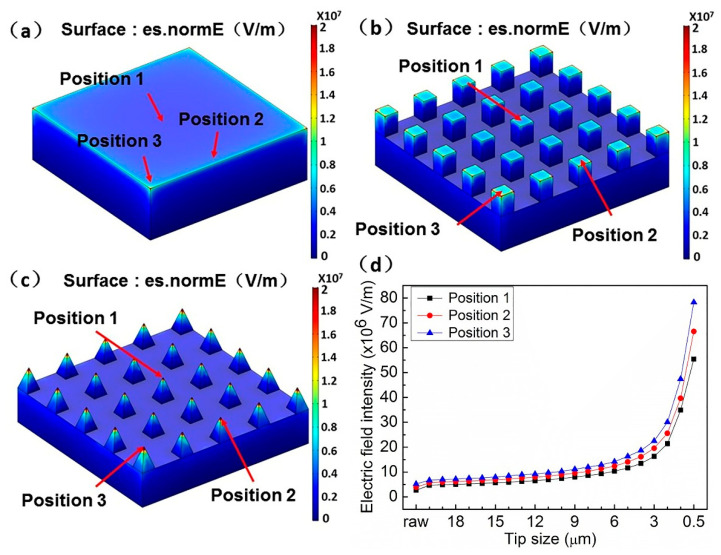
(**a**) The electric field strength distribution of the model of raw VACNT film. (**b**) The electric field strength distribution of the model of the arrays with a tip size of 20 μm. (**c**) The electric field strength distribution of the model of the nanocone arrays with a tip size of 500 nm. (**d**) The electric field strength calculation results of the middle point (Position 1, the center point of the upper surface of the center array corresponds to the center point of the upper surface of VACNT), edge-point (Position 2, the center point of the upper surface of the third array of edges corresponds to the position of the same coordinates of VACNT), and the center point (Position 3, the center point of the upper surface of the corner array corresponds to the same coordinate position of VACNT) of the top surface of the cone arrays of tip size from 0.5–20 μm and the raw VACNT.

A three-dimensional numerical calculation by Comsol Multiphysics software was carried out. According to the calculation results of the three models, the screen effect of the nanocone array with a tip size of 500 nm is the weakest (Figure 5c), which is followed by the arrays of tip size of 20 μm (Figure 5b), and the strongest screening effect is the raw VACNT film (Figure 5a). The nanocone arrays that have a tip size of 500 nm have more regions with higher electric field intensity, which are followed by the arrays with a tip size of 20 μm. According to the actual situation, each carbon nanotube has a certain field screen effect on the surrounding carbon nanotubes, and the strength of the field screen effect is related to their height and the distance between them [38]. The grooves formed by laser processing of the cone arrays with tip sizes of 500 nm and 20 μm help to reduce the field screen effect and make the edge and tip carbon nanotubes obtain higher electric field strength. Moreover, the sharp area is more conducive to the increase in electric field strength. Figure 5d shows the calculation results of the electric field strength at positions 1 (the center point of the upper surface of the center array corresponds to the center point of the upper surface of VACNT), 2 (the center point of the upper surface of the third array of edges corresponds to the position of the same coordinates of VACNT), and 3 (the center point of the upper surface of the corner array corresponds to the same coordinate position of VACNT) of the cone arrays of tip size from 0.5 to 20 μm and the raw VACNT. It shows the increasing trend of the electric field at positions 1, 2, and 3 with the decrease in tip size and their comparison with unprocessed VACNT. It also shows the comparison results at different positions (positions 1, 2, and 3).

The processed sample model has higher field strength at positions 1, 2, and 3, and the field strength tends to increase with the decrease in tip size. At the same time, the field strength at different positions in the same model is also different. Compared with the center position, the number of adjacent carbon nanotubes at the corner position is less and the shielding effect is smaller, so it has higher field strength. However, in the case of the same area, the smaller diameter means that the number of carbon nanotubes is less, which means that the number of emitters is also reduced. The game between the two is the two main factors that determine the field emission performance.

## 4. Results and Discussion

Here, we report a method of patterning VACNT thin film via inexpensive and universal ultraviolet laser ablation. Through the study of the interaction process between laser and VACNT and the reasonable control of this process, the nanocone arrays are fabricated by using a micron light spot. Then, the impacts of this facile patterning were explored with regard to the CNT metrology and the field electron emission properties. The maximum turn-on electric field of a CNT cold cathode has been shown to reduce by over 1 V/μm as a result of UV laser patterning, and the maximum current density increasing by more than 97× (average current of overall area). The 12 h stability test shows that the maximum reduction of the current attenuation rate is more than 20%, with the VACNT samples proving to be increasingly robust and less easily damaged by the strong electric fields during emission. The results of Raman spectroscopy and XPS show that the UV laser-processed VACNTs have more structural defects as well as increased trace elements leading to likely doping during the UV laser processing, which we attribute to the in-air operation of the system. This may be due to the ablation of carbon nanotubes by removing the thermal effect in processing, and high-energy photons tend to directly break the chemical bonds of carbon nanotubes. Some fine carbon nanotube emitters formed by processing may also be helpful for emission. Numerical simulations validated our empirical findings, evidencing that the surface field strength of the processed array can be enhanced by more than one order of magnitude under the same conditions, the field enhancement factor is greatly improved, and the shielding effect is greatly reduced.

In general, this method can easily manufacture a cold cathode electron source with high and stable current density and low turn-on electric field on a large scale at a low cost. It is hoped that the presented facile and inexpensive laser patterning strategy will open up new processing opportunities for emerging nanomaterials-based technologies and in doing so will accelerate the adoption and integration of emerging 1D and 2D nanomaterials in a range of new applications.

## Data Availability

The data presented in this study are available on request from the corresponding author.

## Supplementary Materials

The following are available online at https://www.mdpi.com/article/10.3390/nano11123244/s1, Figure S1: Large aspect ratio array processed by the UV laser. (a) The aspect ratio exceeds 12. (b) The aspect ratio is about 14 (Tilt: 45°), Figure S2: (a) CAD drawings for machining and (b) corresponding processed shape, Figure S3: The simple schematic diagram of field emission test device, Figure S4: (a) For VACNT processed with different frequencies, the number in the figure is the frequency value used (kHz) (20% maximum output power, scanning once, scanning speed 200 mm/s, focusing distance −300 μm), which means that based on the characteristics of the laser, the pulse width and maximum output power will change correspondingly with the change of frequency, (b) is the curve of maximum output power and pulse width with frequency, Figure S5: SEM images of the effect of: (a) Different power (Focal distance: −150 ± 100 μm, scanning times: 1 s, scanning speed: 200 mm/s). (b) Different defocus distance (A positive value is a positive focus and a negative value is a negative focus, in units of μm. power: 2.3 W, scanning times: 1, scanning speed: 200 mm/s) (c) Different scanning times (power: 1.9 W, defocus distance: −150 ± 100 μm, scanning speed: 200 mm/s. Right illustration parameters: frequency: 80 kHz, pulse width: 18.5 ns, power: 2.2 W, speed: 150 mm/s) (d) Different scanning speed (in units of mm/s, power: 2.03 W, Defocus distance: −150 ± 100 μm, scanning speed: 200 mm/s), Figure S6: Original Raman spectra of the raw area and A–O points marked in Figure S4a. In all Raman measurements in this paper, the Raman laser spot is smaller than the tip size of the measured array. (a) Original Raman spectra of the point A marked in Figure S4a. (b) Original Raman spectra of the point B marked in Figure S4a. (c) Original Raman spectra of the point C marked in Figure S4a. (d) Original Raman spectra of the point D marked in Figure S4a. (e) Original Raman spectra of the point E marked in Figure S4a. (f) Original Raman spectra of the point F marked in Figure S4a. (g) Original Raman spectra of the point G marked in Figure S4a. (h) Original Raman spectra of the point H marked in Figure S4a. (i) Original Raman spectra of the point I marked in Figure S4a. (j) Original Raman spectra of the point J marked in Figure S4a. (k) Original Raman spectra of the point K marked in Figure S4a. (l) Original Raman spectra of the point L marked in Figure S4a. (m) Original Raman spectra of the point M marked in Figure S4a. (n) Original Raman spectra of the point N marked in Figure S4a. (o) Original Raman spectra of the point O marked in Figure S4a. (p) Original Raman spectra of the raw area, Figure S7: The ID/IG value of the Raman spectrum of the raw and the point A to point O marked in Figure S4a, Figure S8: (a) The XPS survey scan spectrum and (b) the C1s XPS peaks of the raw sample. (c) The XPS survey scan spectrum and (d) The C1s XPS peaks of another sample (The content ratio of main elements is C:O:N:S = 89.437:9.958:0.455:0.15), Figure S9: The typical picture of other nanocone arrays which have different tip sizes processed by the UV laser. (a–c) The tip size is approximately 2.5 μm. (d–h) The tip size is approximately 5 μm. (i–k) The tip size is approximately 10 μm. (l–n) The tip size is approximately 15 μm, Figure S10: Fine carbon nanotubes on an array processed by the UV laser. (a) Fine emitters on conical arrays and (b) Enlarged view of fine emitter on the side. (c) Fine emitters with more uniform orientation on the rectangular cylinder array and (d) Enlarged view of fine emitter on the side. (e) An array of rectangular cylinders with only small emitters on the side and (f) Enlarged view of fine emitter on the side, Figure S11: Comparison of the morphology of processed and unprocessed samples tested under the same conditions. (a) The raw VACNT before tested. (b–c) The processed Cone array and (d–e) Cone cylinder array with the tips before tested. (f–i) The VACNT after tested, a large area of VACNT was damaged and deep holes appeared during the test. (j–m) The processed cone array after being tested, A small area was damaged during the test, mainly at the edge, and a small part of the top of the array showed the explosive fracture. (n–q) Cone array with tip after tested. Only a few arrays on the edge were damaged during the test. A few arrays changed slightly during the test.
