# Supplementary material for: Nanocone-Shaped Carbon Nanotubes Field-Emitter Array Fabricated by Laser Ablation
Source: Nanomaterials (Basel). 2021 Nov 29;11(12):3244. doi: 10.3390/nano11123244 (PMC8707308; doi:10.3390/nano11123244)
Supplement: Supplementary file 1 [file nanomaterials-11-03244-s001.zip › nanomaterials-1476019-supplementary.pdf]

nanomaterials

MDPI

Supplementary Materials

# Nanocone-Shaped Carbon Nanotubes Field-Emitter Array Fabricated by Laser Ablation

**Jiuzhou Zhao [1,2], Zhenjun Li [2,3], Matthew Thomas Cole [4], Aiwei Wang [2,5], Xiangdong Guo [2,5], Xinchuan Liu [2], Wei Lyu [1,2], Hanchao Teng [2,5], Yunpeng Qv [2,5], Guanjiang Liu [2], Ke Chen [2,5], Shenghan Zhou [2,5], Jianfeng Xiao [2], Yi Li [1,6,*], Chi Li [2,5,*] and Qing Dai [2,5,*]**

[1] Tianjin Key Laboratory of Molecular Optoelectronic Sciences, Department of Chemistry, School of Science, Tianjin University & Collaborative Innovation Center of Chemical Science and Engineering (Tianjin), Tianjin 300072, China; zhaojz2020@nanoctr.cn (J.Z.); lvw0817@163.com (W.L.)

[2] CAS Key Laboratory of Nanophotonic Materials and Devices, CAS Key Laboratory of Standardization and Measurement for Nanotechnology, CAS Center for Excellence in Nanoscience, National Center for Nanoscience and Technology, Beijing 100190, China; lizhenjun@nanoctr.cn (Z.L.); wangaw2020@nanoctr.cn (A.W.); guoxd@nanoctr.cn (X.G.); liuxc2019@nanoctr.cn (X.L.); tenghc2019@nanoctr.cn (H.T.); quyp2020@nanoctr.cn (Y.Q.); liugj20101120@163.com (G.L.); chenke@nanoctr.cn (K.C.); zhoushenghan@nanoctr.cn (S.Z.); xiaojf2020@nanoctr.cn (J.X.)

[3] GBA Research Innovation Institute for Nanotechnology, Guangzhou 510700, China

[4] Department of Electronic and Electrical Engineering, University of Bath, Bath BA27AY, UK; mtc47@bath.ac.uk

[5] Center of Materials Science and Optoelectronics Engineering, University of Chinese Academy of Sciences, Beijing 100049, China

[6] Joint School of National University of Singapore and Tianjin University, Fuzhou International Campus, Tianjin University, Binhai New City, Fuzhou 350207, China

* Correspondence: liyi@tju.edu.cn (Y.L.); lichi@nanoctr.cn (C.L.); daiq@nanoctr.cn (Q.D.)

## Complete experimental details

VACNT films were synthesized on silicon (type n, 0.001–0.005 Ω·cm, <100>) substrate by PECVD (Plasma enhanced chemical vapor deposition).The acetone and Isopropyl alcohol cleaned N-type <100> Si substrates (450 ± 10 µm thick) were first spin-coated with a photoresist (s1813 at 3000 r/min for 60 s) in order to create a square outline pattern (side length 2 mm) which defines the extent of the experimentation. Following resist development, a bilayer catalyst of 10/1 nm Fe/Al were then thermally evaporated (Chongwen Technology Co., Ltd., Tianjin, China; OHMIKER-50B) at a rate of ~0.1 nm/s, with the residual photoresist subsequently removed with acetone and isopropanol sonication rinses in a heated water bath for 10 min at 60 °C. Catalyst coated samples were then transferred into the PECVD (Aixtron NanoInstruments Co., Ltd., Herzogenrath, Germany; Black Magic 2) vacuum chamber and then pumped to a base pressure of $3 \times 10^{-2}$ mbar. VACNT thin films were then grown under 20 sccm $C_2H_2$ (Beijing Millennium capital Gas Co., Ltd., Beijing, China; ≥ 99.5%) 500 sccm $H_2$ (Beijing Millennium capital Gas Co., Ltd., Beijing, China; ≥ 99.5%) and 200 sccm $N_2$ (Beijing Millennium capital Gas Co., Ltd.; ≥ 99.5%). at a growth pressure of 10 mbar at 520–580 °C (thermal ramp rate 5 °C/s) for 20 min. Following growth samples were cooled under $N_2$ flow until they reached a temperature of < 200 °C when they were removed from the growth system. A growth time of 10–20 min produced VACNTs with heights of 400–700 µm, The VACNT films were characterized by scanning electron microscope (SEM) (Hitachi, Ltd. Tokyo, Japan; SU8220,10 kV) and transmission electron microscopy (TEM) (FEI, Ltd. Hillsborough, Oregon, USA; Tecnai G2 20 S-TWIN).

Following CNT growth, macroscale 2 mm × 2 mm square monoliths of VACNT thin films were realized, ready for subsequent UV laser processing. Femtosecond laser processing has been demonstrated elsewhere though commonly at different wavelengths such as 800 nm and 1064 nm, however in the present work nanosecond ultraviolet laser processing platform (λ = 355 nm, Suzhou Delong laser Co., Ltd., Suzhou, China; FP-D-DZS-001) was used. The patterning system consists of an integrated computer-controlled

stepper stage capable of minimum x, y step sizes of 1 μm, and a maximum scan range of 100 mm. The UV laser (Suzhou Beilin Laser Co., Ltd., Suzhou, China; sp355-10) is 15 μm in diameter. AutoCAD (Version 2018, Autodesk, Inc. San Rafael, California, USA) software was used in this work to create DGW files required by the laser patterning system. The CAD drawings outline the path of the laser in two dimensions. The coordination of various parameters determines the width, depth, shape, continuity and smoothness of the ablation groove, which together with the CAD drawings determines the shape of the VACNT created by the laser.

UV laser ablation patterning affords a facile approach to the realization of otherwise time-consuming and complex patterns within CNT thin films. It has been shown that micro and macro scale geometries within CNT arrays enhanced their field electron emission performances. Thus, here we explored the use of the developed patterning technique to engineering new electron emission sources. Cubic arrays with sides of 20 μm and spacing of 40 μm were first fabricated on the VACNT film, then we used this as a benchmark to continuously reduce the size of the tip, and fabricated the cone arrays with the tip size of about 15 μm, 10 μm, 5 μm, 2.5 μm and 500 nm on the VACNT film which were all initially 608 ± 20 μm thick.

The effects of different laser processing parameters on the carbon nanotubes have been explored, including Raman spectroscopy (Renishaw plc, London, UK; Renishaw in Via plus) as a function of laser power, which were also were characterized by X-ray photoelectron spectroscopy (XPS, Thermo Fisher Technology (China) Co., Ltd, Shanghai, China; ESCALAB250Xi) to explore the impacts of carbon ablation on the VACNTs chemical composition and crystallography before and after processing.

Field emission measurements were carried in a custom-built vacuum chamber evacuated to a base pressure of $< 1 \times 10^{-7}$ mbar with a turbo molecular pump. Measurements were conducted in diode mode. To minimize anode-induced arcing, the anode was formed from a 5 mm thick mechanically polished stainless steel plate (surface roughness), with the cathode formed from either a processed or unprocessed VACNT thin film on the stainless steel substrate, The distance between cathode and anode was 462 ± 20 μm. A 10 kV DC power supply (Tianjin Dongwen high voltage power supply Co., Ltd., Tianjin, China; DW-P103-20) was attached via a 500 kΩ ( ± 1%) ballast resistor and connected with the cathode via a vacuum feed through. The field emission current was measured by a microcomputer (Tianjin Dongwen high voltage power supply Co., Ltd., Tianjin, China; DW-P103-20ACDE), and the high voltage power supply bias control automatic using a High voltage power supply control software (Tianjin Dongwen high voltage power supply Co., Ltd. Tianjin, China;). Voltages were swept from 0–10 kV with $\Delta V = 50$ V and a dwell/step time of 1 s. A schematic diagram of the test equipment is in the Figure S3.

A three-dimensional numerical calculation by Comsol Multiphysics software (Version 5.5, Comsol, Inc. Stockholm, Sweden) was carried out to verify the influence of field screen effect on carbon nanotubes cold cathode.

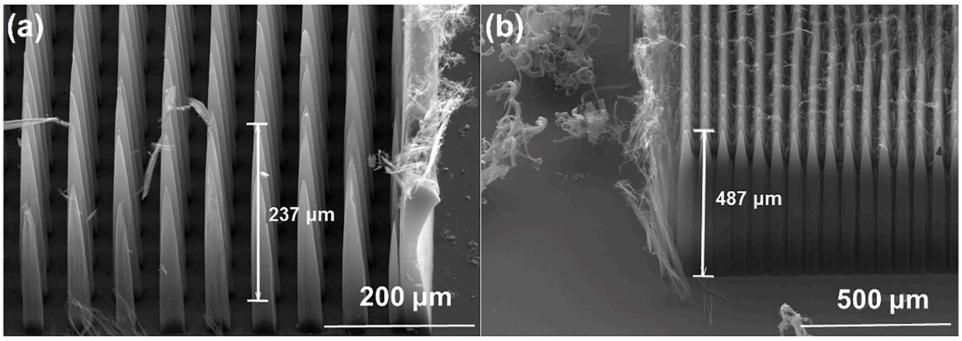


**Figure S1.** Large aspect ratio array processed by the UV laser. (**a**) The aspect ratio exceeds 12. (**b**) The aspect ratio is about 14 (Tilt: 45°).

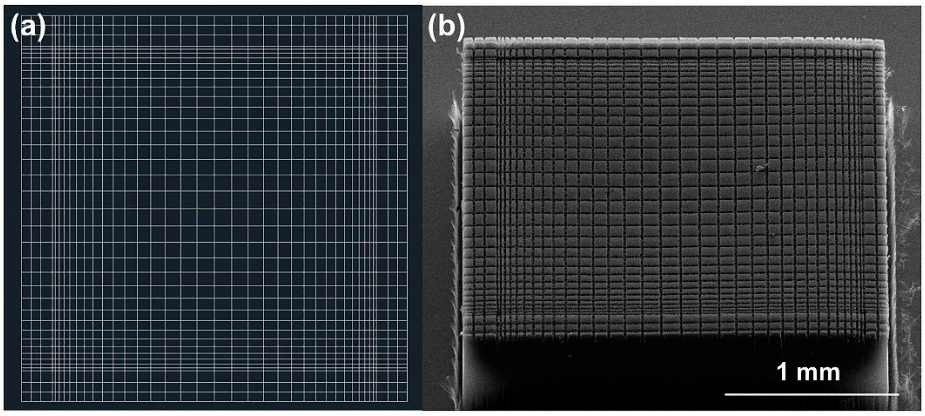


**Figure S2.** (**a**) CAD drawings for machining and (**b**) corresponding processed shape.

CAD software was used in this work to create DGW files required by the laser patterning system.

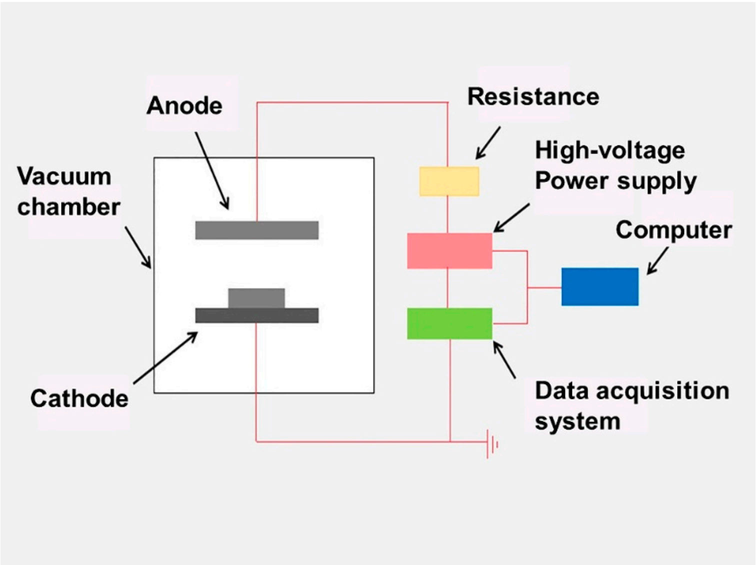


**Figure S3.** The simple schematic diagram of field emission test device.

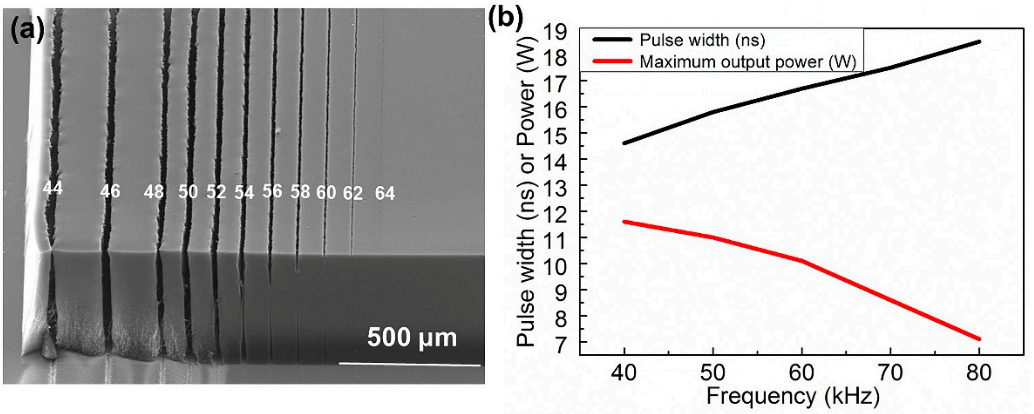


**Figure S4.** (**a**) For VACNT processed with different frequencies, the number in the figure is the frequency value used (kHz) (20% maximum output power, scanning once, scanning speed 200 mm/s, focusing distance −300 μm), which means that based on the characteristics of the laser, the pulse width and maximum output power will change correspondingly with the change of frequency, (**b**) is the curve of maximum output power and pulse width with frequency.

Since for all experiments the laser frequency was fixed at 60 kHZ with a pulse width at 16.7 ns, with other parameters (laser power, defocus distance, scanning speed and scanning times) then being investigated. In order to show the effect of processing parameters on the VACNTs more intuitively, a side of the VACNT we selected has a small slope angle with the horizontal plane, rather than being vertical, and then gradually change one of these parameters, such as power, scanning time, scanning speed and focal distance.

Figure S5a shows a typical SEM image of laser ablated VACNT as a function of laser power (1.19–3.91 W). The depth and width of the ablated trenches are found to increase significantly with an increase in laser power, the volume of the ablated VACNTs increases significantly. From 1.86 W to 3.11 W, the width of the groove has a 270% variation. When the power density is low (< 4.65 × 105 W/cm$^2$), the ablated groove tends to form a tapered shape with uneven width (The focus is below the VACNT upper surface.). Typically such widths have shown up to 260% variation in the groove width across the entire depth of the ablated groove. For high power densities (> 4.65 × 105 W/m$^2$), the VACNT was penetrated, and the shape of the ablated groove is more regular, adopting more rectangle profiles. Figure 5b shows an SEM image that depicts the influence of different focal distances on VACNT ablation. The position of point 0 in the figure is the known focal point. We define the focus above the upper surface of VACNT as positive focus, which is represented by a positive value, and the focus below the upper surface of VACNT as negative focus, which is represented by a negative value. We find that the depth, width and shape of the ablated grove are especially sensitive to the focal distance as the probe optical energy density and the greatest when the spot is the smallest.

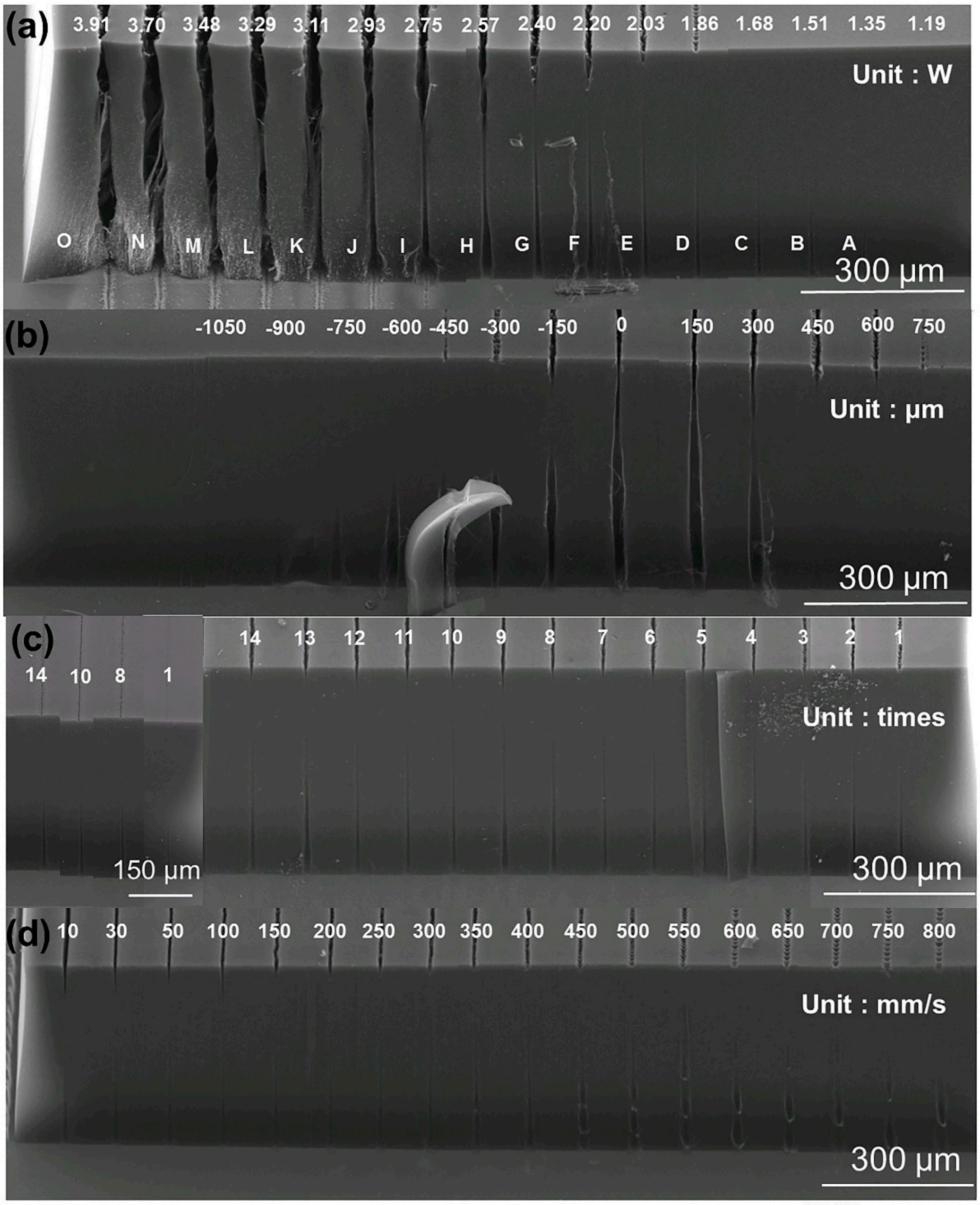


**Figure S5.** SEM images of the effect of: (**a**) Different power(Focal distance: −150 ± 100 μm, scanning times: 1 s, scanning speed: 200 mm/s.) (**b**) Different defocus distance(A positive value is a positive focus and a negative value is a negative focus, in units of μm. power: 2.3 W, scanning times: 1, scanning speed: 200 mm/s) (**c**) Different scanning times(power: 1.9 W, defocus distance: –150 ± 100 μm, scanning speed: 200 mm/s. Right illustration parameters: frequency: 80 kHz, pulse width: 18.5 ns, power: 2.2 W, speed:150 mm/s) (**d**) Different scanning speed (in units of mm/s, power: 2.03 W, Defocus distance: −150 ± 100 μm, scanning speed: 200 mm/s).

During the process from the far positive defocusing distance to the focal point, the width of the groove on the upper surface of VACNT becomes narrower and the depth increases (for example, from 750 μm to 0, the groove width increased by about 240% and the depth increased by at least 48.7 × ), and the shape of the groove changes from a conical structure to increasingly rectangular. In the process of moving from positive to a negative focus, the observed change in the VACNTs ablated grooves is opposite to that of moving from positive to negative focus, and the height of the groove near the end of the silicon wafer shows a downward trend, which indicates that the laser energy is highly divergent at these distant positions, which results in low energy densities that are below the ablation

threshold (≈50 mJ·cm$^{-2}$) of the VACNTs. Therefore, for VACNT with different heights, it is necessary to precisely adjust the height of the sample table in order to retain probe focus and ensure depth uniformity during ablation.

Figure S5c shows the effect of probe scanning time. When the laser scans the sample surface once, the depth of the ablative groove is shallow, and the surface presents discontinuous and uneven pits. By increasing the number of scans, the ablative groove becomes deeper and the surface becomes smoother. (The number of scans increased from 1 to 6, and the groove depth increased by about 600%.) When the number of scans is > 8, the extent to which the ablated pattern changes becomes negligibly small with each additional pass. The right illustration shows the study of scanning times in another set of parameters. It can be seen that when the scanning times increase from 1 to 8, 10 and 14, the change of groove depth and width is slower than that in the left figure, which also shows that the cooperation of various parameters in laser processing is extremely important. Figure S5d shows the influence of scanning speed on VACNT similar to the influence of scanning times on VACNT. As the scanning speed decreases, the depth of the ablation groove increases, and the ablated area gradually becomes continuous and smooth. At the same time, the width of the ablation groove slightly increases, and the shape of the ablation groove gradually becomes tapered.

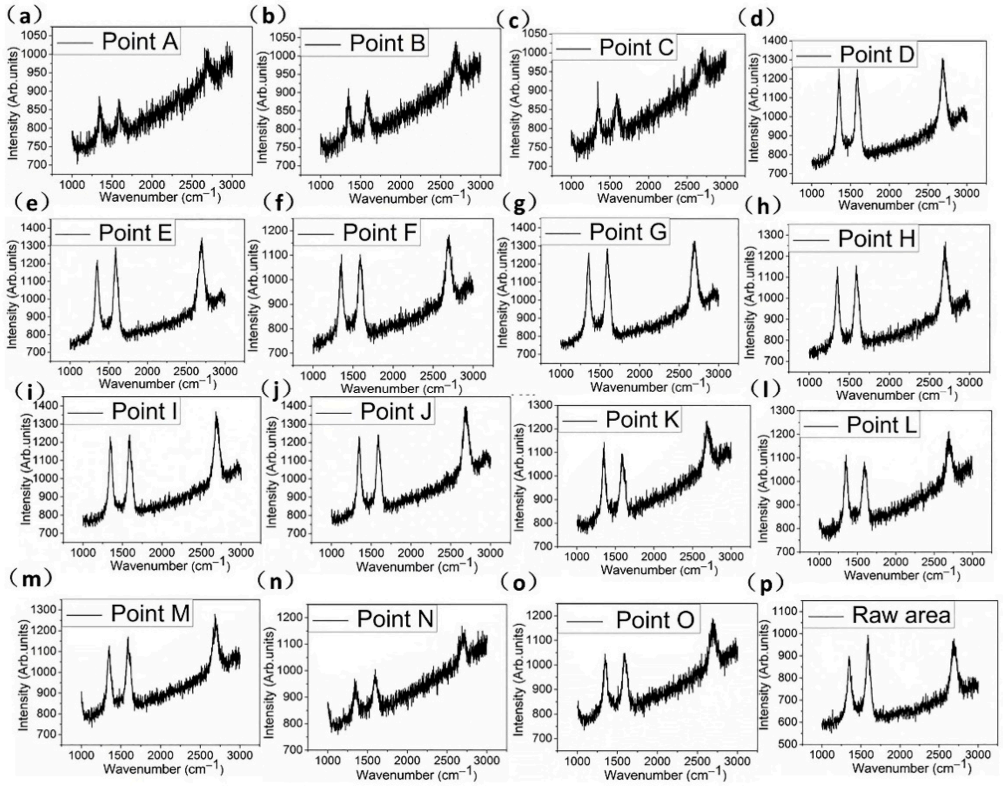


**Figure S6.** Original Raman spectra of the raw area and A–O points marked in Figure S4a. In all Raman measurements in this paper, the Raman laser spot is smaller than the tip size of the measured array. (**a**) Original Raman spectra of the point A marked in Figure S4a. (**b**) Original Raman spectra of the point B marked in Figure S4a. (**c**) Original Raman spectra of the point C marked in Figure S4a. (**d**) Original Raman spectra of the point D marked in Figure S4a. (**e**) Original Raman spectra of the point E marked in Figure S4a. (**f**) Original Raman spectra of the point F marked in Figure S4a. (**g**) Original Raman spectra of the point G marked in Figure S4a. (**h**) Original Raman spectra of the point H marked in Figure S4a. (**i**) Original Raman spectra of the point I marked in Figure S4a. (**j**) Original Raman spectra of the point J marked in Figure S4a. (**k**) Original Raman spectra of the point K marked in Figure S4a. (**l**) Original Raman spectra of the point L marked in Figure S4a. (**m**) Original Raman spectra of the point M marked in Figure S4a. (**n**) Original Raman spectra of the point N marked in

Figure S4a. (**o**) Original Raman spectra of the point O marked in Figure S4a. (**p**) Original Raman spectra of the raw area.

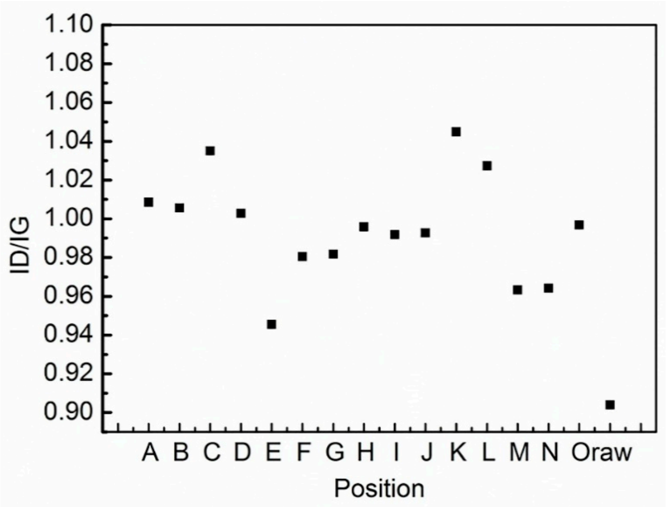


**Figure S7.** The ID/IG value of the Raman spectrum of the raw and the point A to point O marked in Figure S4a.

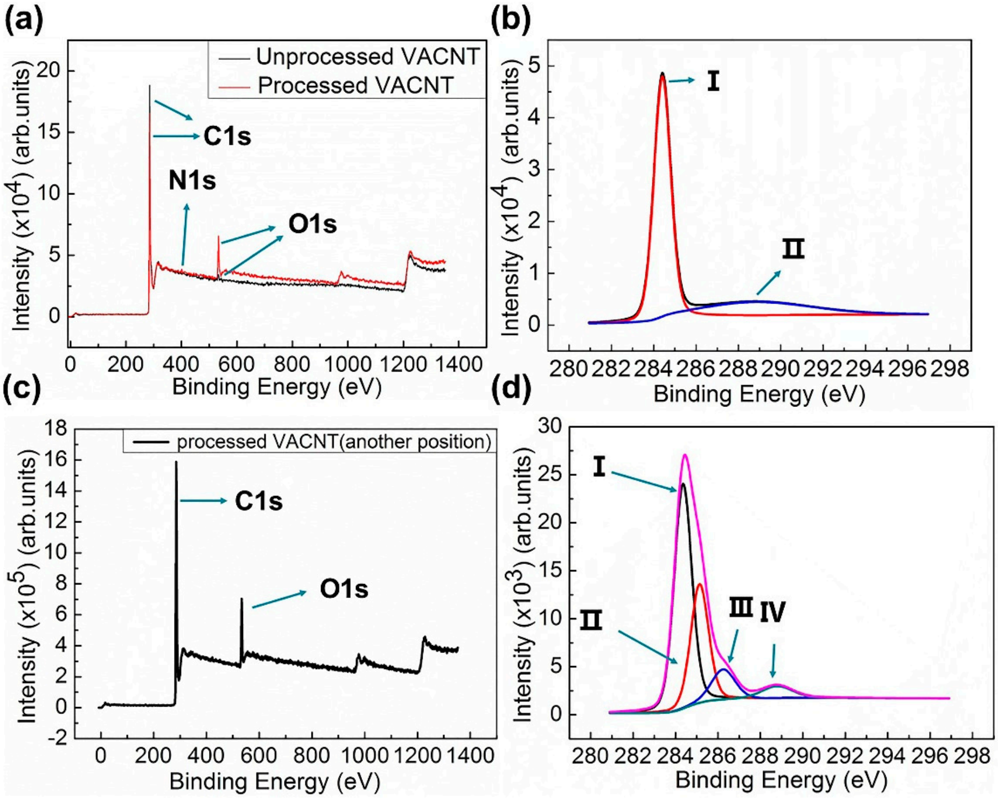


**Figure S8.** (**a**) The XPS survey scan spectrum and (**b**) the C1s XPS peaks of the raw sample. (**c**) The XPS survey scan spectrum and (**d**) The C1s XPS peaks of another sample (The content ratio of main elements is C:O:N:S = 89.437 : 9.958 : 0.455:0.15).

Figure S8b shows the C1s peaks of the raw VACNT. Peak I is attributed to graphite-like C–C bonds and peak IV is attributed to C–O type bonds.

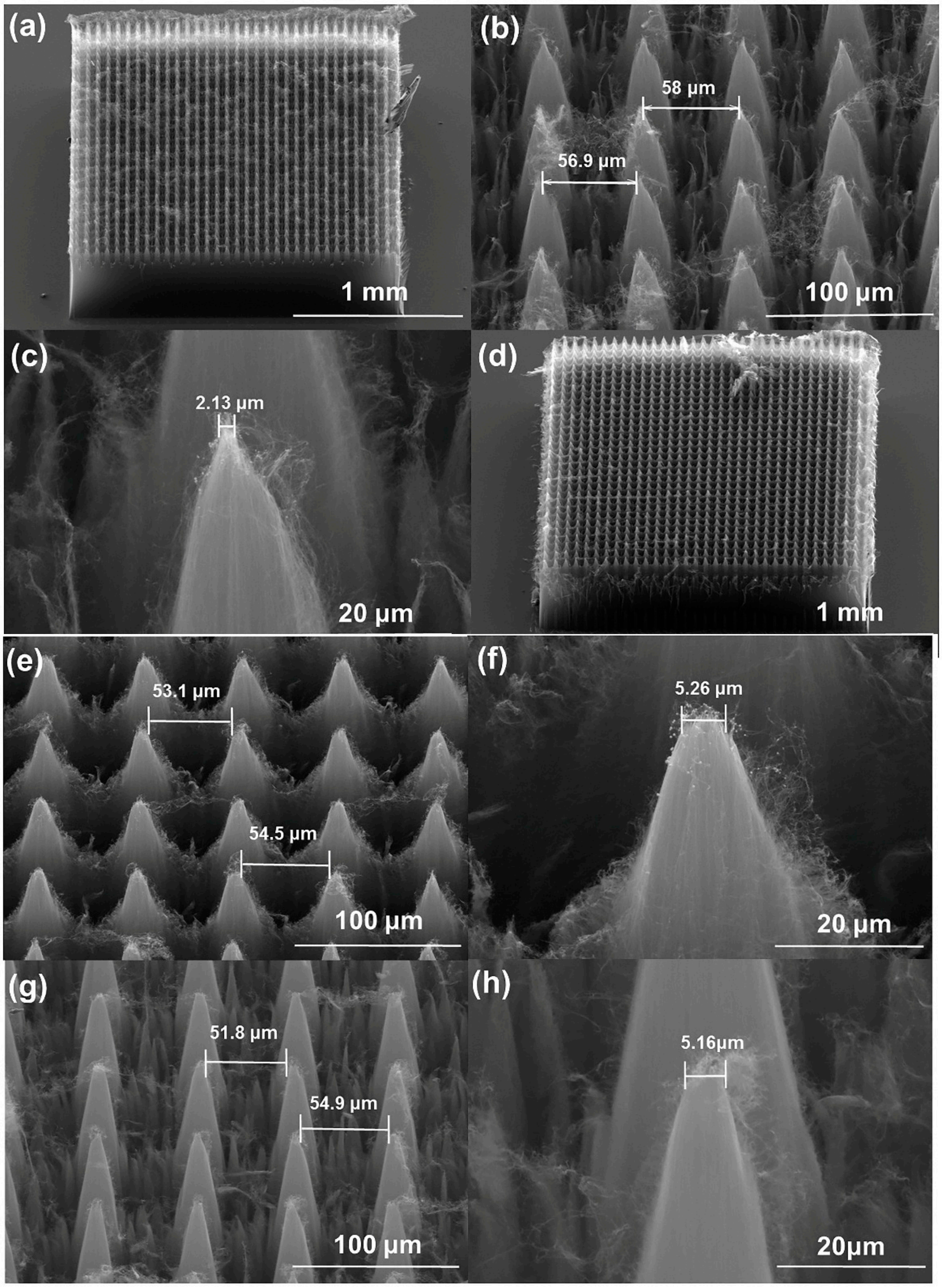
(a)
1 mm
(b)
58 μm
56.9 μm
100 μm
(c)
2.13 μm
20 μm
(d)
1 mm
(e)
53.1 μm
54.5 μm
100 μm
(f)
5.26 μm
20 μm
(g)
51.8 μm
54.9 μm
100 μm
(h)
5.16μm
20μm

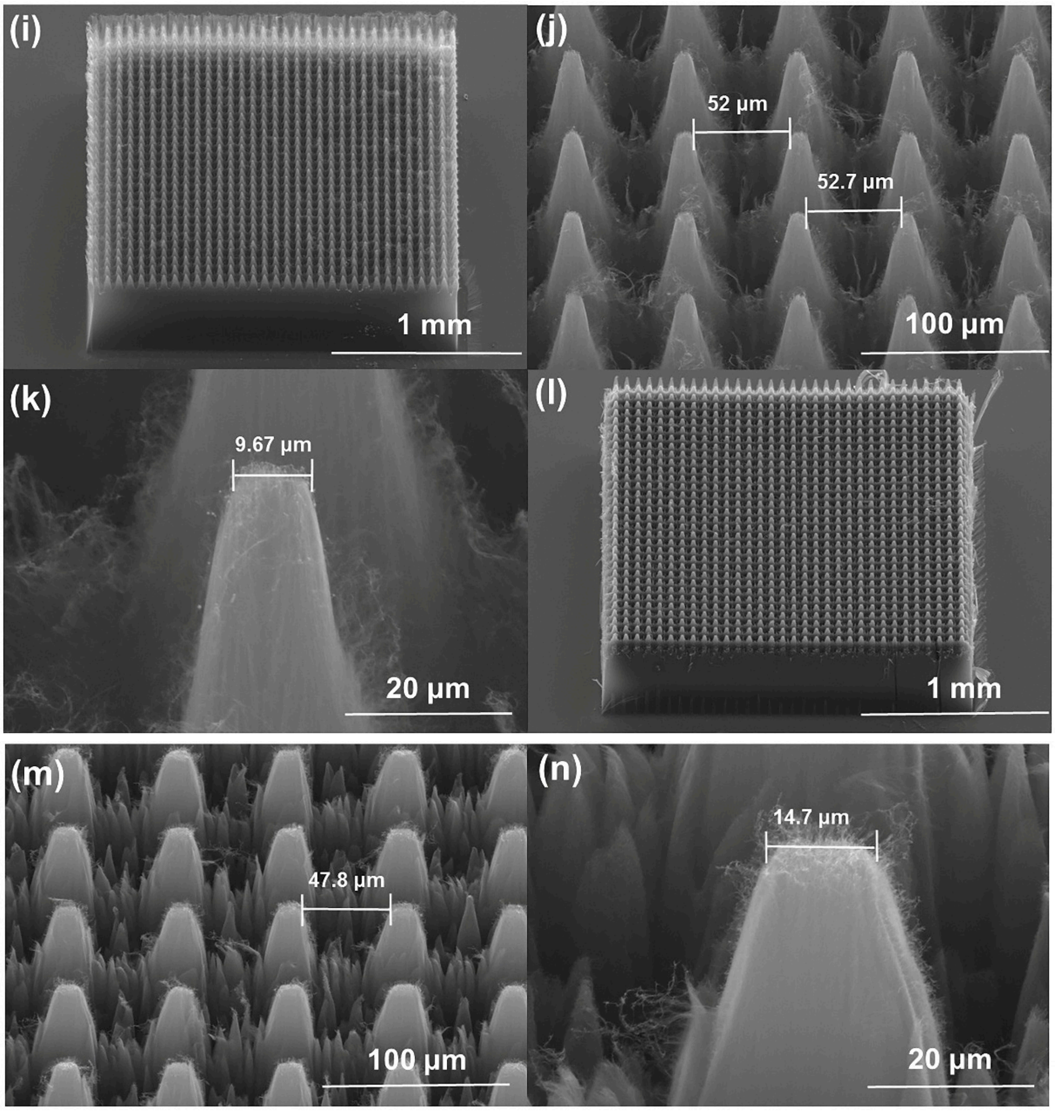


**Figure S9.** The typical picture of other nanocone arrays which have different tip sizes processed by the UV laser. (**a**–**c**) The tip size is approximately 2.5 μm. (**d**–**h**) The tip size is approximately 5 μm. (**i**–**k**) The tip size is approximately 10 μm. (**l**–**n**) The tip size is approximately 15 μm.

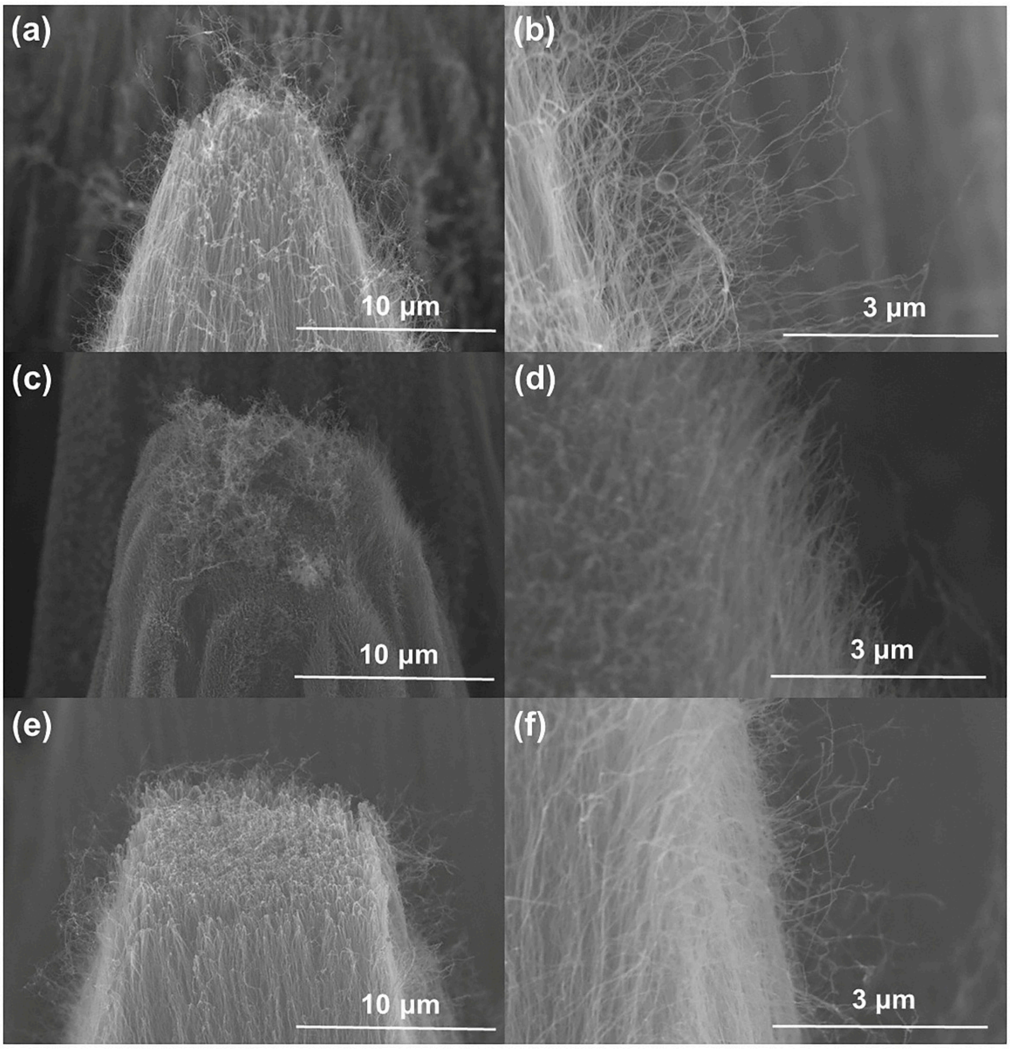


**Figure S10.** Fine carbon nanotubes on an array processed by the UV laser. (**a**) Fine emitters on conical arrays and (**b**) Enlarged view of fine emitter on the side. (**c**) Fine emitters with more uniform orientation on the rectangular cylinder array and (**d**) Enlarged view of fine emitter on the side. (**e**) An array of rectangular cylinders with only small emitters on the side and (**f**) Enlarged view of fine emitter on the side.

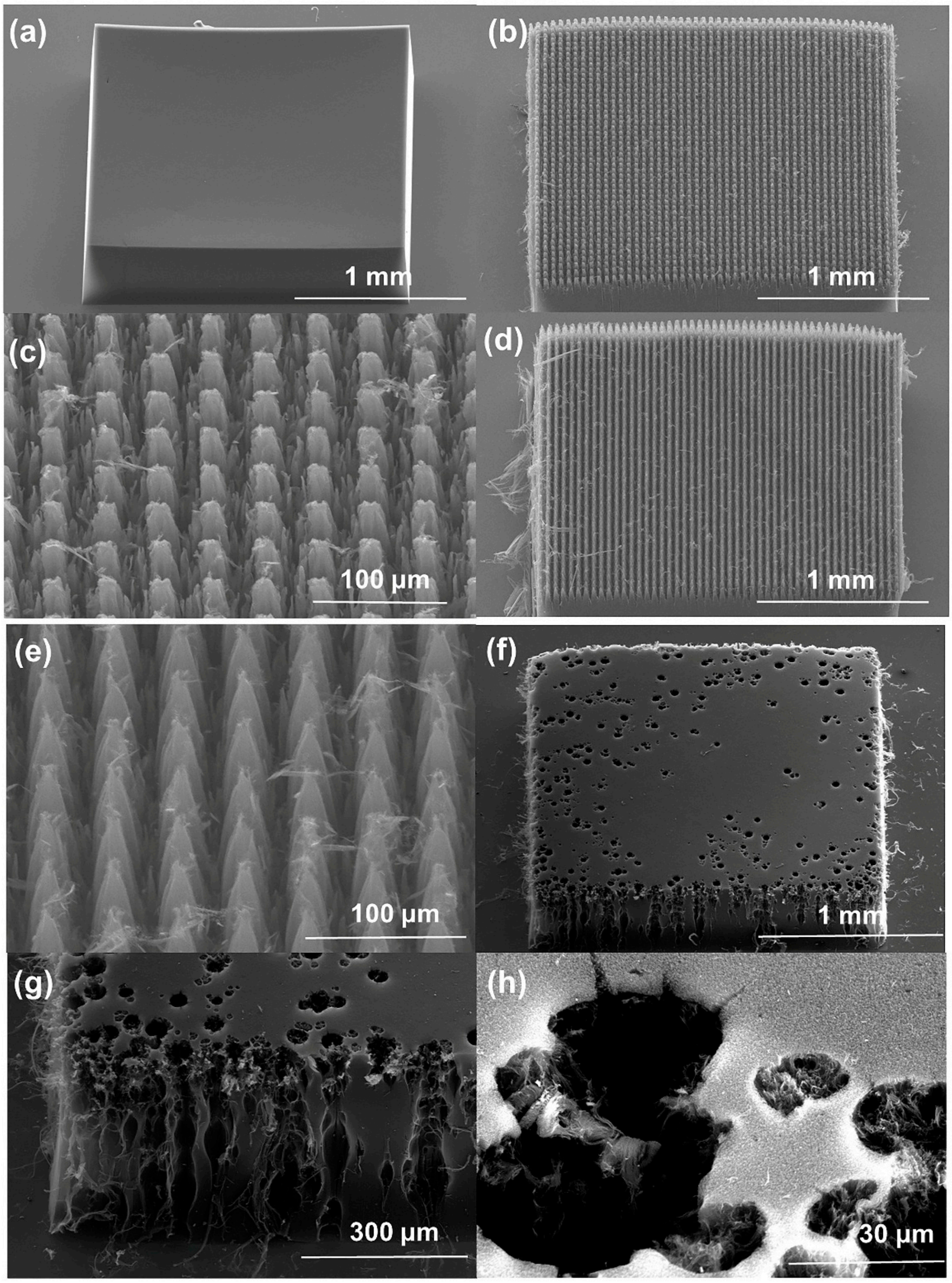
(a)
1 mm
(b)
1 mm
(c)
100 µm
(d)
1 mm
(e)
100 µm
(f)
1 mm
(g)
300 µm
(h)
30 µm

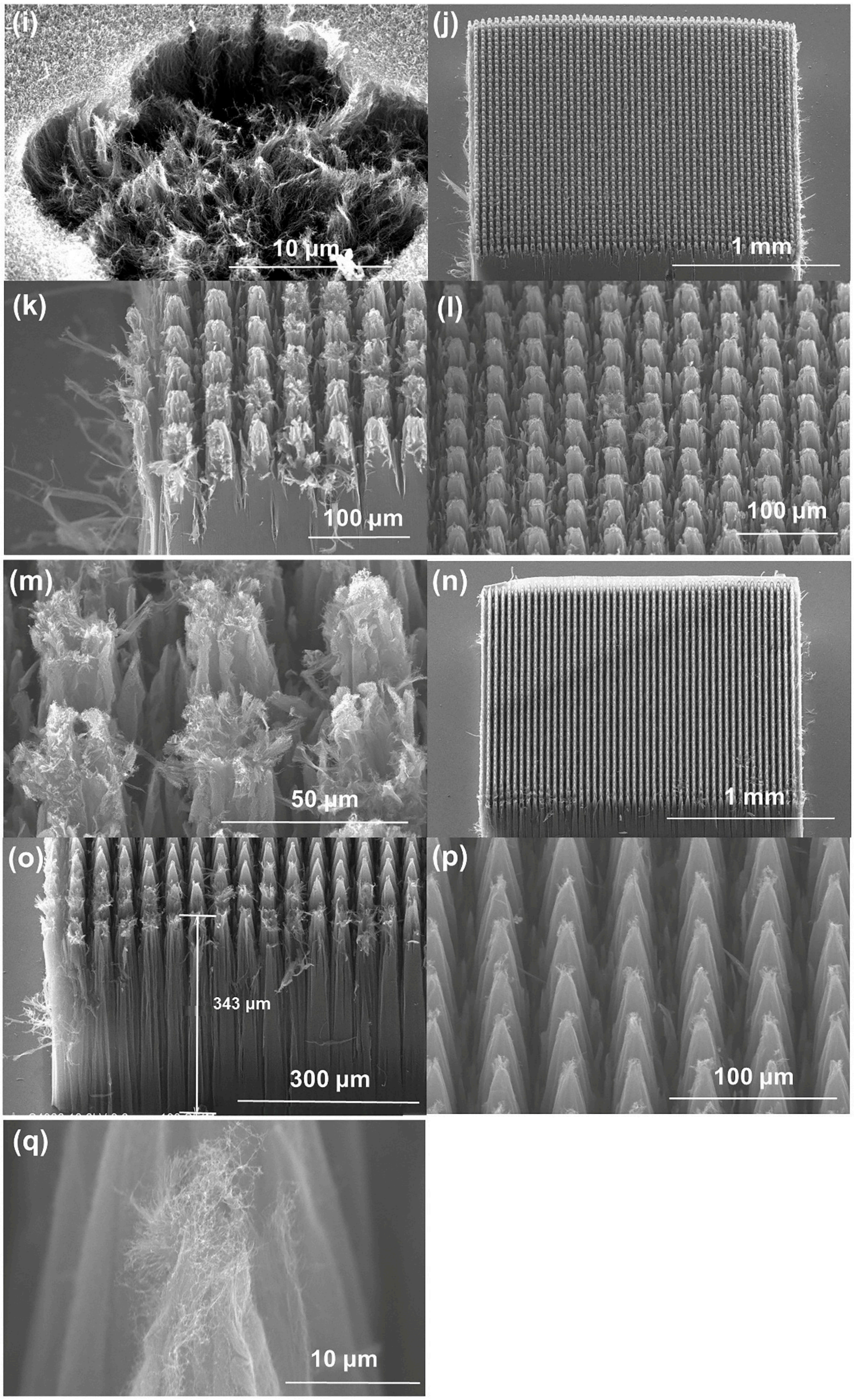


**Figure S11.** Comparison of the morphology of processed and unprocessed samples tested under the same conditions. (**a**) The raw VACNT before tested. (**b,c**) The processed Cone array and (**d,e**) Cone cylinder array with the tips before tested. (**f–i**) The VACNT after tested, a large area of VACNT was damaged and deep holes appeared during the test. (**j–m**) The processed cone array after being

tested, A small area was damaged during the test, mainly at the edge, and a small part of the top of the array showed the explosive fracture. (**n–q**) Cone array with tip after tested. Only a few arrays on the edge were damaged during the test. A few arrays changed slightly during the test.

We suspect that the damage of the sample during the test is partly due to the effect of voltage, and partly due to the large amount of heat generated by the continuous current during the test, which is easy to burn the carbon nanotubes.

**Simulation details**

In the process of modeling, due to the limited computing power of the computer, if the calculation is carried out according to the actual size, the calculation results will not be accurate enough or the calculation will fail, this is because the surface of the carbon nanotube cathode needs to be divided into very small grids to ensure the calculation accuracy, which occupies most of the computer performance, so we simplified the calculation model.

The model of the raw sample was reduced by 7.7× on the basis of real size and was built into a cuboid with a side length of 260 μm and a height of 80 μm. Correspondingly, the model of the arrays with a side length of 20 μm was established as a 5 × 5 cuboid arrays which have a side length of 20 μm and a height of 20 μm vertically arranged on a VACNT with a side length of 260 μm and a height of 60 μm, the side length of the whole model is also 260 μm and the height is 80 μm, which is consistent with that of the unprocessed sample. Then parametric scanning was carried out on this basis to make its side length from 20 μm gradually change to 500 nm, and the scanning step is 1 μm. In this way, we get the calculation results of array samples with different tip diameters from 500 nm to 20 μm, including, of course, the array samples with side lengths of 500 nm, 5 μm, 10 μm, 15 μm and 20 μm that we have built and tested. In order to make the calculation results as close as possible to the real situation, the models of silicon wafer and cathode tray used to support silicon wafer are also constructed. In the simulation calculation, the carbon nanotubes were regarded as good conductors, the dielectric constant was set to 1, the distance between the anode plate and cathode plate was set to 462 μm, which was consistent with the value during the field emission test. The cathode plate, silicon wafer and carbon nanotube model surface were set to be grounded according to the actual test process, the anode plate had a potential of 2000 V. In order to get more accurate results, the mesh should be divided as small as possible when the computer computing power allows. Here, we set the maximum mesh on the surface of the carbon nanotube model as no more than 400 nm and the minimum mesh as 15 nm.
